# Progressive Thickening of the Fingers in Adolescence: A Case Report of a Rare Entity

**DOI:** 10.7759/cureus.101083

**Published:** 2026-01-08

**Authors:** Diana L Alfaro-Ponce, Guadalupe Maldonado-Colin, Veronica Martinez Garcia, Lucia Achell Nava

**Affiliations:** 1 Dermatology, Universidad Nacional Autonoma de Mexico, Mexico City, MEX; 2 Dermatology, Centro Medico Nacional 20 de Noviembre, ISSSTE, Mexico City, MEX

**Keywords:** adolescent, dermatology, digital fibromatosis, pachydermodactyly, subcutaneous tissue

## Abstract

Pachydermodactyly (PPD) is a rare, benign digital fibromatosis that predominantly affects adolescent males and can mimic inflammatory or rheumatologic disorders. We report a case of a 16-year-old male with a six-year history of painless, progressive periarticular thickening of the hands and feet, primarily involving the second through fourth interphalangeal joints. The patient reported repetitive mechanical exposure associated with basketball and video game use. Laboratory studies, imaging, and a skin biopsy were performed to exclude alternative diagnoses. Histopathology demonstrated fibrous dermal hyperplasia, epidermal acanthosis with hyperkeratosis, and increased stromal mucins, consistent with transgressive PPD. Conservative management with topical corticosteroids resulted in stabilization of the disease. Early recognition of this entity is essential to avoid unnecessary investigations and inappropriate treatment, particularly in adolescents, for whom cosmetic concerns may significantly affect quality of life.

## Introduction

Pachydermodactyly (PPD) is a rare, benign form of digital fibromatosis characterized by painless periarticular soft tissue thickening of the fingers without underlying joint involvement [1,2]. Its true incidence remains unknown, and it is considered an uncommon condition in routine clinical practice; the most recent reviews report approximately 150 published cases worldwide [2]. PPD predominantly affects adolescent males and typically manifests around puberty, most commonly during the early to mid-teen years [2]. The condition was first described in 1973 by Bazex et al. as digital fibromatosis with cutaneous hyperplasia [1], and later termed pachydermodactyly by Verbov in 1975, who recognized it as a distinct clinical entity [2]. Repetitive mechanical stimulation or chronic microtrauma affecting the periarticular skin of the proximal interphalangeal joints has been proposed as a major contributing factor in its pathogenesis [2,3]. We report a case of bilateral transgrediens PPD confirmed by clinical evaluation and histopathology, adding to the limited existing literature and highlighting the importance of recognizing this benign fibromatous disorder in the differential diagnosis of digital soft-tissue enlargement without joint damage.

## Case presentation

**Figure 1:**
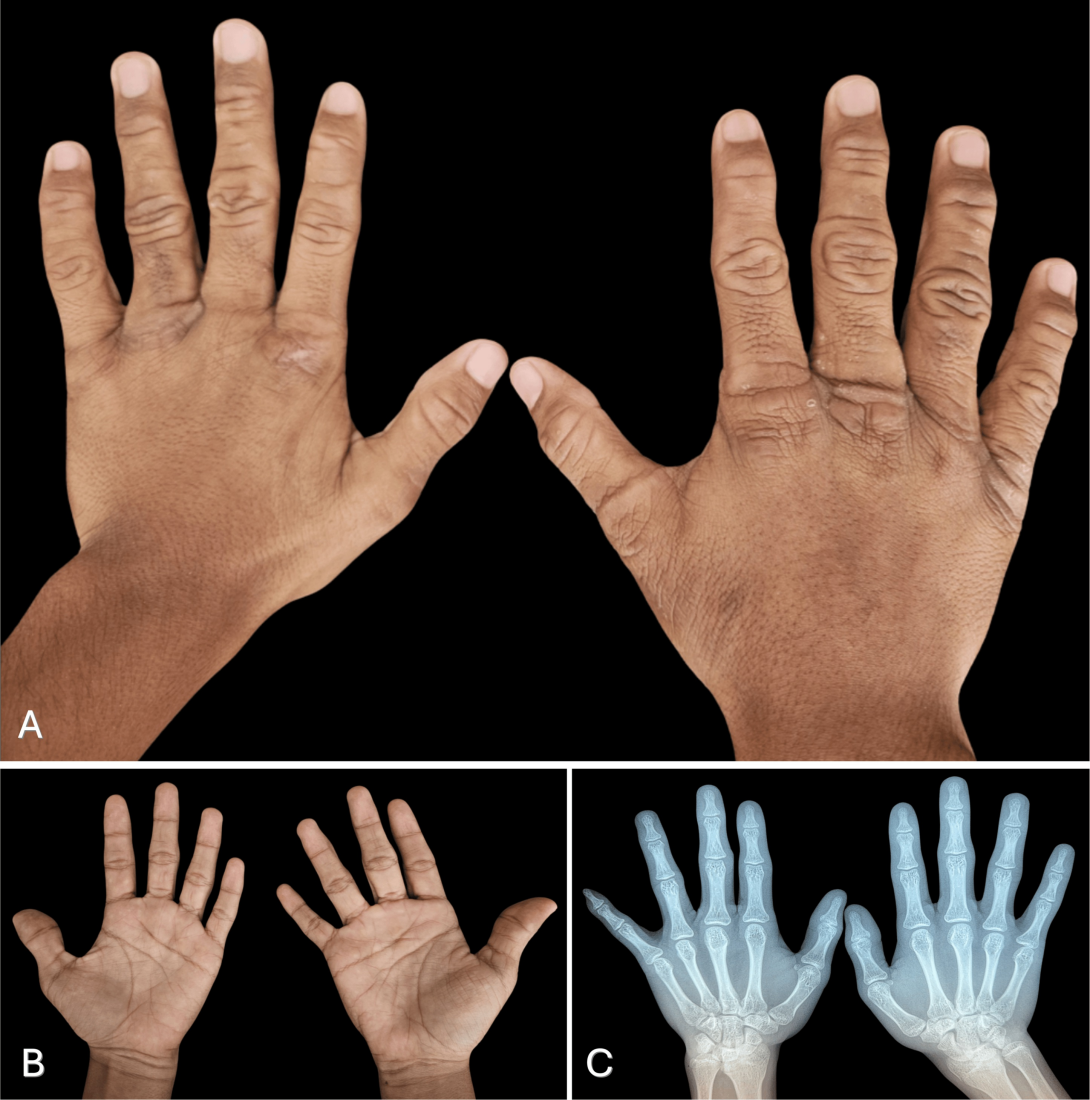
Clinical image and X-ray of hands (A-B) Involvement of the dorsal region of the hands, with a predominance in the interphalangeal region consisting of thickening, hyperpigmentation, and hyperlianity. (C) Radiographs demonstrated increased soft tissue volume without evidence of bone involvement

A 16-year-old male high school student with no significant congenital or acquired medical history was referred to the dermatology department for evaluation of diffuse bilateral hand thickening that had been present for approximately six years. Physical examination revealed a symmetric dermatosis of the hands and feet, predominantly involving the second, third, and fourth interphalangeal joints of both hands (Figures 1A-1B). The lesions were characterized by lateral soft tissue thickening, hyperkeratosis, velvety hyperpigmentation, and pronounced hyperlinearity of the affected skin. The patient denied pain, pruritus, fever, morning stiffness, or other systemic or articular complaints. He reported regular basketball practice and frequent video game use, both considered potential sources of repetitive mechanical stress.

The diagnostic workup focused on excluding deposition disorders and connective tissue diseases. Laboratory evaluation, including complete blood count and renal and hepatic function tests, showed no abnormalities. Inflammatory markers, including erythrocyte sedimentation rate and C-reactive protein, were within normal limits. Rheumatologic testing revealed negative antinuclear antibodies, rheumatoid factor, human leukocyte antigen B27, extractable nuclear antigen antibodies, anti-topoisomerase I, and anti-Jo-1 antibodies. Plain radiographs of the hands demonstrated increased soft tissue volume without evidence of bone or joint abnormalities (Figure 1C). Evaluation by the rheumatology service concluded that the patient did not meet the criteria for any rheumatologic disease.

**Figure 2:**
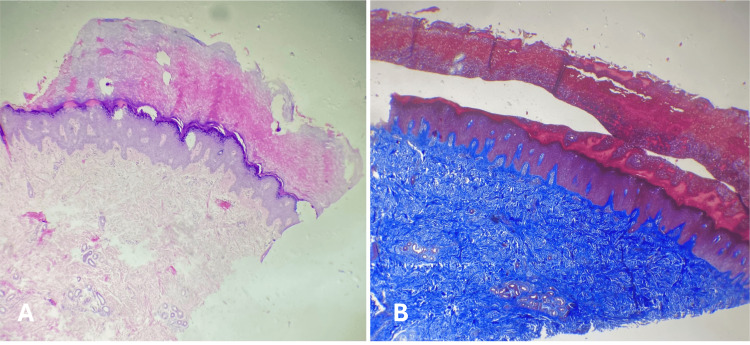
Fragment of the skin (A) Hyperkeratosis, acanthosis, and fibrous hyperplasia in superficial dermis (hematoxylin-eosin stain, x4). (B) Fibrous hyperplasia in the dermis (Masson's trichrome staining, x10)

Due to the persistence of the cutaneous findings, a skin biopsy was obtained from a lesion on the left hand (Figures 2A-2B). Histopathological examination revealed fibrous dermal hyperplasia, epidermal acanthosis with elongation of the dermal papillae, and marked hyperkeratosis. Special stains were negative for fungal elements and amyloid deposition (Congo red). Alcian blue staining demonstrated increased stromal mucins, and Masson trichrome staining highlighted dermal fibrous proliferation.

Based on the absence of symptoms, lack of morning stiffness, painless joint motion, lateral rather than circumferential swelling, normal laboratory results, and radiographic findings limited to soft tissue involvement, differential diagnoses such as connective tissue diseases and juvenile idiopathic arthritis were excluded. A diagnosis of transgrediens PPD was therefore established.

Treatment with topical corticosteroids was initiated, and the patient was placed under clinical follow-up. After one year of observation, no progression of the lesions was noted. The patient currently remains under conservative management with emollient therapy. Given the benign nature and stability of the condition, the prognosis is favorable.

## Discussion

PPD is a rare, benign fibromatous condition that predominantly affects adolescent males and typically presents around puberty [2,3]. Clinically, it is characterized by painless periarticular soft tissue thickening, most commonly involving the proximal interphalangeal joints of the second through fifth fingers [2]. Although the exact etiology remains uncertain, repetitive mechanical microtrauma is consistently identified as a major contributing factor, particularly in individuals engaged in activities that require frequent hand use, such as sports, playing musical instruments, or prolonged computer and video game use [3,4]. While associations with genetic conditions, including tuberous sclerosis and Ehlers-Danlos syndrome, have been reported, most cases are sporadic and lack systemic involvement [3,4].

Chen et al. proposed diagnostic criteria that include (1) absence of symptoms, (2) absence of morning stiffness, (3) painless joint motion without tenderness, (4) lateral rather than circumferential finger swelling, (5) normal laboratory findings, and (6) radiographic findings limited to soft tissue swelling without bone or joint abnormalities [5]. All of these criteria were met in the present case. Histopathologically, PPD typically shows epidermal hyperkeratosis and acanthosis, increased collagen deposition, fibroblast proliferation, and variable dermal mucin accumulation, supporting its classification as a noninflammatory fibromatous disorder [2,3].

Bardazzi et al. classified PPD into five clinical subtypes: classic, localized (monopachydermodactyly), transgrediens, familial, and PPD associated with tuberous sclerosis [2,6]. The transgrediens subtype is defined by extension beyond the interphalangeal joints, with involvement of the dorsum of the hands and metacarpophalangeal regions [2,3,6]. Based on the pattern of clinical involvement, our patient was categorized within this subtype. Differential diagnoses include knuckle pads, rheumatoid arthritis, and juvenile idiopathic arthritis [2,3,7]. These conditions were excluded in our patient on the basis of normal laboratory results, absence of joint involvement on imaging, and supportive histopathological findings. Because of its rarity and clinical overlap with inflammatory disorders, PPD is frequently underrecognized, which may lead to unnecessary diagnostic procedures and treatments.

Management is primarily conservative and focuses on avoiding repetitive mechanical trauma and addressing associated behavioral factors when present [2]. Topical or intralesional corticosteroids can be considered in selected cases, particularly when cosmetic concerns are prominent [2,3]. Surgical treatment is rarely required and is reserved for refractory cases. Overall, PPD is associated with an excellent prognosis.

## Conclusions

PPD is a rare, benign disorder that can mimic inflammatory or rheumatologic conditions. Recognition of its typical clinical distribution, benign course, normal laboratory and imaging results, and supportive histopathological features is essential to prevent misdiagnosis and unnecessary interventions. Early identification and increased awareness are particularly important in adolescents, for whom cosmetic changes to the hands may significantly affect quality of life.
